# Current Trends in the Management of Gastroesophageal Reflux Disease: A Review

**DOI:** 10.5402/2012/391631

**Published:** 2012-07-11

**Authors:** Sylvester Chuks Nwokediuko

**Affiliations:** Gastroenterology Unit, Department of Medicine, University of Nigeria Teaching Hospital Ituku/Ozalla, PMB, Enugu 01129, Nigeria

## Abstract

Gastroesophageal reflux disease (GERD) is a chronic disorder of the upper gastrointestinal tract with global distribution. The incidence is on the increase in different parts of the world. In the last 30 to 40 years, research findings have given rise to a more robust understanding of its pathophysiology, clinical presentation, and management. The current definition of GERD (The Montreal definition, 2006) is not only symptom-based and patient-driven, but also encompasses esophageal and extraesophageal manifestations of the disease. The implication is that the disease can be confidently diagnosed based on symptoms alone. Nonerosive reflux disease (NERD) remains the predominant form of GERD. Current thinking is that NERD and erosive reflux disease (ERD) are distinct phenotypes of GERD rather than the old concept which regarded them as components of a disease spectrum. Non erosive reflux disease is a very heterogeneous group with significant overlap with other functional gastrointestinal disorders. There is no gold standard for the diagnosis of GERD. Esophageal pH monitoring and intraluminal impedance monitoring have thrown some light on the heterogeneity of NERD. A substantial proportion of GERD patients continue to have symptoms despite optimal PPI therapy, and this has necessitated research into the development of new drugs. Several safety concerns have been raised about chronic use of proton pump inhibitors but these are yet to be substantiated in controlled studies. The debate about efficacy of long-term medical treatment compared to surgery continues, however, recent data indicate that modern surgical techniques and long-term PPI therapy have comparable efficacy. These and other issues are subjects of further research.

## 1. Introduction

Gastroesophageal reflux disease (GERD) is a common chronic disorder prevalent in many countries [1]. Apart from the economic burden of the disease and its associated impact on quality of life [2–5], it is the most common predisposing factor for adenocarcinoma of the esophagus. As a consequence of the irritation caused by the reflux of acid and bile, adenocarcinoma may develop in these patients, representing the last of a sequence that starts with the development of GERD and progresses to metaplasia (Barrett's esophagus), low-grade dysplasia, high-grade dysplasia, and adenocarcinoma. Although there has been a decrease in the incidence of squamous cell cancers, the rate of esophageal adenocarcinoma has increased rapidly, and this has been traced to the advent of obesity epidemic, GERD and Barrett's esophagus [6, 7].

Over the years, several issues have emerged regarding the definition, classification, natural history and treatment of GERD, and complications associated with its treatment. This paper focuses on some of these evolving issues. Recent studies, limited to English language, were identified via PubMed searches (1990–2011) with the search terms GERD, NERD, prevalence, incidence, epidemiology, and management. Recent reviews on epidemiology and management were also examined for appropriate references.

## 2. Definition

Until recently, there were many definitions of GERD. The lack of a gold standard for diagnosis made it difficult to adopt a satisfactory definition. The first ever global consensus definition was published in 2006. According to that document, GERD is defined as “a condition which develops when the reflux of stomach contents causes troublesome symptoms and/or complications” [1]. Based on this definition, GERD can be classified into 2 syndromes: esophageal and extraesophageal syndromes (Table 1). This definition recognizes that GERD can be diagnosed in primary care on the basis of symptoms alone without additional diagnostic testing. This approach is appropriate for most patients and does not use unnecessary resources. Symptoms reach a threshold where they constitute disease when they are troublesome to patients and affect their functioning during usual activities of living. This patient-centered approach to diagnosis includes asking patients how their symptoms affect their everyday lives.

Heartburn and regurgitation are the characteristic symptoms of GERD. Heartburn is defined as a burning sensation in the retrosternal area. Regurgitation is defined as the perception of flow of refluxed gastric contents into the mouth or hypopharynx. These symptoms are sufficiently descriptive to be diagnostic. Esophageal and extraesophageal symptoms and syndromes that form part of the framework of GERD also include chest pain, sleep disturbances, cough, hoarseness, asthma, and dental erosions (Table 1) [1].

## 3. Epidemiology

Gastroesophageal reflux disease is now the most common upper gastrointestinal disease in the western countries, with 10% to 20% of the population experiencing weekly symptoms [4, 8]. In Asia, the prevalence has been variously reported but is generally lower (2.3% by Wong et al. and 6.2% by Chen et al.) [9, 10]. Population-based survey studies indicate that the prevalence is rising [5]. Possible explanations for this include aging population, the obesity epidemic (and associated changes in diet or physical activity), and changes in sleep pattern [11].

A limited number of studies have reported GERD and its complications to be rare in Africa [12]. However, a recent study of Nigerian medical students showed a prevalence of 26.3% [13]. Nonerosive reflux disease (NERD) accounts for over 60% of cases of GERD in Nigeria [14].

## 4. Classification

Gastroesophageal reflux disease is broadly classified into 2 groups on the basis of endoscopy findings: having esophageal mucosal damage (erosive esophagitis and Barrett's esophagus) and no mucosal damage (endoscopy-negative reflux disease or nonerosive reflux disease, NERD).

Traditionally, GERD had been approached as a spectrum disease, with NERD at the mild end and complicated GERD (stricture, Barrett's esophagus, or adenocarcinoma) at the other end of the spectrum. However, emerging evidence indicates that the vast majority of NERD and erosive esophagitis (ER) patients remain within their respective GERD groups throughout their lifetime [15, 16]. This new paradigm proposes that the genetic makeup of each individual subject exposed to similar environmental factors may ultimately determine the specific phenotypic presentation of GERD. In other words, GERD phenotypes once determined remain true to form [15, 16].

Nonerosive reflux disease (NERD) patients have been subclassified into 3 types on the basis of the results of 24-hour pH evaluation: 

*Type 1*: Patients who demonstrate an abnormal acid exposure time in a manner similar to those with erosive esophagitis [17].

*Type 2*: Patients with a normal acid exposure time, but with symptoms and reflux events that are significantly correlated, suggesting acid hypersensitivity. This is also referred to as “the hypersensitive esophagus” [17–19].

*Type 3*: Patients with typical reflux symptoms, but normal pH studies, and no correlation between symptoms and acid exposure. Within this group are 2 subgroups; namely: those who respond to proton pump inhibitor therapy and those who do not respond. The latter subgroup represents functional heartburn (according to Rome III guideline) [20].

A combination of conventional esophageal pH monitoring and intraluminal impedance monitoring now offers the opportunity to detect acid and non-acid reflux and their association with symptoms [21]. Using this technique, NERD patients with normal pH studies were found to have a positive symptom association for acid reflux in 15% but also a positive association for non-acid reflux in 12% of patients [22]. These findings have led to the narrowing down of the proportion of patients who were otherwise labeled as presenting with functional heartburn, leading to the identification of a new subgroup of patients whose symptoms are due to reflux other than acid; a subgroup of patients with nonacid reflux disease (NARD) or weakly acidic reflux disease (WARD).

## 5. Risk Factors

There is a potential genetic component to the development of GERD and perhaps Barrett's esophagus [23]. In the US, although the frequency of GERD symptoms does not differ between Caucasians and African Americans, the latter group have a persistently lower risk of esophagitis [24]. In a study from Johannesburg, of the 216 consecutive Barrett's esophagus patients only 5% were black despite the ratio of Blacks to Whites in the city being 5 : 1 [5].

There is evidence to suggest that age and male sex are associated with a higher incidence of esophagitis [25–27]. Obese subjects are 2.5 times more likely to have GERD than those with normal body mass index (BMI) [28]. Several other researchers have reported similar relationship between body mass and GERD [29, 30]. Alcohol consumption and the presence of a hiatus hernia are risk factors for GERD and esophagitis [25, 31]. The presence and size of a hiatal hernia are associated with a more incompetent LES, defective peristalsis, more severe mucosal damage, and increased acid exposure [32]. A Japanese study identified cigarette smoking and alcohol as risk factors for GERD [33]. In Nigeria, increased consumption of cola and coffee by medical students in order to stay awake to read for examinations was associated with an increased prevalence of GERD [14]. One study showed that an initial diagnosis of either GERD or irritable bowel syndrome raised the risk of a subsequent diagnosis of the other three fold [34]. Gastroesophageal reflux disease is frequently found in patients with connective tissue disease, especially scleroderma [35], as well as patients with chronic obstructive airway disease [34]. In addition, a number of common drugs and hormonal products have been associated with GERD. These include anticholinergics, benzodiazepines, calcium channel blockers, dopamine, nicotine, nitrates, theophylline, estrogen, progesterone, glucagon, and some prostaglandins. Heartburn is a very common gastrointestinal manifestation of pregnancy.

## 6. Pathophysiology

Reflux is a normal physiologic occurrence and is produced most often by transient relaxation of the lower esophageal sphincter (LES). In patients with GERD, these transient relaxations occur more frequently than normal. The basal pressure of this sphincter is 10–45 mmHg. The crural diaphragm and gastric sling fibres provide structural support and contribute to LES pressure and competence. The ability of the LES to maintain a tone higher than structures proximal and distal is a result of spikes of calcium influx that are mediated by excitatory cholinergic neurons [36]. Higher intracellular calcium levels are present in the resting LES compared with nonsphincteric esophageal muscle. Other defects of the LES that may contribute to GERD include a chronically hypotensive LES and the effects of a hiatal hernia.

Under normal situations, endogenous defense mechanisms either limit the amount of noxious material that is introduced into the esophagus or rapidly clear the material from the esophagus so that symptoms and esophageal mucosal irritation are minimized. Examples of such defense mechanisms include actions of the LES and normal esophageal motility. When the defense mechanisms are defective or become overwhelmed so that the esophagus is bathed in acid or bile-containing fluid for prolonged periods, GERD can be said to exist.

The esophagus, LES, and stomach can be likened to a simple plumbing circuit [37]. The esophagus functions as an anterograde pump, the LES as a valve, and the stomach as a reservoir. The abnormalities that contribute to GERD can stem from any component of the system. A dysfunctional LES allows reflux of large amounts of gastric juice. Delayed gastric emptying can increase volume and pressure in the reservoir until the valve mechanism is overwhelmed, leading to GERD. Esophageal defense mechanisms include esophageal clearance and mucosal resistance. Esophageal clearance has a mechanical arm (esophageal peristalsis) and a chemical component (saliva), both of which limit the amount of time the esophagus is exposed to refluxed gastric juice. 

Transient relaxation of the LES can be caused by foods (coffee, alcohol, chocolate, fatty and meals), medications (beta-blockers, nitrates, calcium channel blockers, anticholinergics), hormones (progesterone), and nicotine.

Regarding the effect of hiatal hernia, not all patients with hiatal hernias have symptomatic reflux. In the presence of a hiatal hernia, the LES may migrate proximally into the chest and lose its abdominal high-pressure zone (HPZ), or the length of the HPZ may decrease. The diaphragmatic hiatus may be widened by a large hernia, which impairs the ability of the crura to function as an external sphincter. Also the gastric contents may be trapped in the hernia sac and reflux proximally into the esophagus during relaxation of the LES. Reduction of the hernias and crural closure result in the restoration of an adequate intra-abdominal length of esophagus and recreating the HPZ.

## 7. Diagnosis

There is no gold standard for the diagnosis of GERD. Endoscopy is positive in only about 40% of cases [14]. Furthermore, the evaluation of antireflux therapies is based on resolution of symptoms and this suffers greatly from subjectivity. The Society of American Gastrointestinal Endoscopic surgeons (SAGES) Practice Guidelines stipulates that the diagnosis of GERD can be confirmed if at least one of the following conditions exists: a mucosal break seen on endoscopy in a patient with typical symptoms, Barrett's esophagus on biopsy, a peptic stricture in the absence of malignancy, or positive pH-metry [38]. This definition obviously excludes patients with NERD who are negative on pH-metry. Therefore, an objective diagnostic tool with acceptable sensitivity and specificity remains an unmet need for clinicians and researchers.

### 7.1. Clinical Diagnosis

Heartburn and regurgitation are characteristic symptoms of the typical reflux syndrome [9]. The typical reflux syndrome can be diagnosed on the basis of characteristic symptoms without diagnostic testing [1], provided that alarm symptoms have been excluded. Alarm symptoms are symptoms which raise a strong suspicion of malignant disease or complication. They include vomiting, gastrointestinal bleeding, anemia, abdominal mass, unexplained weight loss, and progressive dysphagia.

Over the years, several symptom-based diagnostic questionnaires have been developed to help primary care physicians in making provisional categorization of patients presenting with upper abdominal complaints and in the selection of patients with reflux symptoms for empirical treatment. The original reflux disease questionnaire developed by Carlsson et al. [39] and a modified version of it [40] have proved to be useful in this respect.

### 7.2. Radiology

This has a low sensitivity and specificity for the diagnosis of erosive esophagitis. It has no place in the diagnosis of NERD.

### 7.3. Endoscopy

This has a high specificity but low sensitivity as over 60% of patients with GERD actually have NERD [14]. In future, new imaging procedures are likely to shed more light on cases that were hitherto classified as NERD by standard white light endoscopy. Such emerging procedures include high-resolution magnification endoscopy, chromoendoscopy, narrow-band imaging, and confocal endomicroscopy [41–43].

### 7.4. Histology

Various histological lesions have been described in NERD. These include dilated intercellular spaces (DIS) [44], basal cell hyperplasia [45], papilla elongation [46], intraepithelial eosinophils [47], and neutrophils [48], with varying sensitivities and specificities. Zentilin et al. [49] proposed a scoring system that takes multiple possible histologic abnormalities into account. Using a receiver operator characteristic curve analysis, a score of 2 was identified as optimal cut-off value for separating GERD patients from controls. A recent study of Nigerian patients with NERD showed a high degree of intraepithelial neutrophil infiltration of the esophageal mucosa; a finding that may be related to the relative rarity of Barrett's esophagus in Nigerians, and indeed black patients [50]. Despite the diagnostic potential of histology, the widespread use of histopathology in clinical practice is hampered by the need for standardization of biopsy and microscopy techniques.

### 7.5. Proton Pump Inhibitor (PPI) Test

In this test, a short trial of PPI to determine if the patient is going to have symptom relief is carried out. Significant symptom improvement suggests GERD. False positive and false negative results can occur in this test. If the patient's history is typical for uncomplicated GERD, an initial PPI trial (including lifestyle modification) is appropriate [51]. This is the position of the American Gastroenterological Association. The Asia-Pacific Consensus on the management of GERD also favors this approach [52].

### 7.6. Manometry

In patients with persistent reflux symptoms despite PPI therapy and normal findings on endoscopy a further evaluation with manometry is indicated to identify alternative diagnosis, such as motor esophageal abnormalities. Manometry helps to analyze the function and the peristaltic activity of the body of the esophagus and the lower esophageal sphincter (LES) prior to antireflux surgery. However manometry is not indicated for confirming a suspected diagnosis of GERD. It is mainly used to establish the diagnosis of dysphagia in cases in which a mechanical obstruction (e.g., stricture) cannot be found. It is also indicated for the preoperative assessment of candidates for antireflux surgery, to exclude achalasia or ineffective peristalsis [53]. Moreover, manometry serves to localize the LES for subsequent pH monitoring for documentation of abnormal esophageal acid exposure.

### 7.7. Ambulatory pH Monitoring

Patients with NERD who do not respond to medications are best evaluated by ambulatory pH monitoring. The test should be performed-off therapy if the diagnosis is under question but should be performed-on therapy if one is trying to determine the adequacy of treatment. The wireless pH radiotelemetry capsule eliminates the need for the uncomfortable nasogastric tube and increases diagnostic yield by allowing for longer monitoring. Ambulatory esophageal pH monitoring is based upon the duration of time the intraesophageal pH is less than 4, with normal defined as less than 4% over a 24-hour period [54]. Up to 50% of patients with NERD have a normal 24-hour pH monitoring study.

Esophageal impedance pH monitoring is a very promising technique. Multichannel intraluminal impedance monitoring with pH sensor (MII-pH) can detect all types of reflux (acidic, weakly acidic, and weakly alkaline). This test measures the resistance of electrical conductivity of the esophageal content, thus detecting any change of esophageal pH due to the presence of liquid or gas reflux [55, 56].

## 8. Treatment

The goals of treatment include relief of symptoms, healing of esophagitis, prevention of recurrence, and prevention of complications. The principles of treatment include lifestyle modifications and control of gastric acid secretion using drugs or surgical treatment with corrective antireflux surgery.

### 8.1. Lifestyle/Dietary Modifications

These are considered the first line of treatment. They include weight loss (for patients who are overweight); avoiding alcohol, chocolate, citrus juice, tomato-based products, peppermint, coffee, and onion. Other measures include avoiding large meals, decreasing fat intake, cessation of smoking, elevation of head of the bed, and avoiding recumbency for 3 hours postprandial [57]. Although there are no randomized trials to test the efficacy of these measures, most gastroenterologists are of the opinion that it is reasonable to employ them. Pregnant women who have GERD should be offered lifestyle modification as first-line therapy.

### 8.2. Antacids/Alginates

These are effective in symptom relief [58–60] and should be taken after each meal and at bed time.

### 8.3. Acid Suppressive Therapy

Currently, acid suppressive therapy forms the mainstay of GERD treatment [61]. Histamine 2 receptor antagonists (H2RAs) can decrease gastric acid secretion after a meal and are better than antacids [62]. however, they are not efficacious in the healing of esophagitis and maintenance therapy with standard doses of H2RAs cannot prevent relapses [63]. Today they are used for the treatment of milder forms of the disease and for on-demand therapy, especially for nocturnal symptoms [64].

Proton pump inhibitors (PPI) are the most potent type of acid suppressants. They are substituted benzimidazoles that irreversibly bind the H^+^K^+^ATPase, the final step in gastric acid secretion [65]. Several trials and reviews have shown the superiority of PPIs over H2RAs in the treatment of reflux esophagitis [61, 62, 66, 67]. For patients with NERD, resolution of symptoms with PPIs is inferior to the response in erosive esophagitis as only 61% of patients experience resolution of heartburn, which is still better than 40% reported for H2RAs [68, 69]. 

Clinical experience shows that 20–30% of patients with GERD continue to have persistent reflux symptoms even while taking PPI daily [70] and one quarter of patients report the use of additional over-the-counter therapies to aid in symptom control [71]. Putative mechanisms for failure of PPI treatment include compliance, improper dosing time, weakly acidic reflux, duodenogastroesophageal reflux (DGER), delayed gastric emptying, esophageal hypersensitivity, eosinophilic esophagitis, nocturnal reflux, residual acid reflux, reduced PPI bioavailability, and psychological comorbidity [72, 73].

Prokinetic agents are somewhat effective but only in patients with mild symptoms; other patients usually require additional acid-suppressing medications such as PPIs. Metoclopramide is a commonly used member of this group. Domperidone has the advantage of less extrapyramidal effects. Long-term use of prokinetic agents may have serious, even potentially fatal complications and should be discouraged. Randomized controlled trials provide moderate-quality evidence that prokinetic drugs improve symptoms in patients with reflux esophagitis and low-quality evidence that they have impact on endoscopic healing [74].

### 8.4. Maintenance Therapy

Recurrence of esophagitis is substantially reduced in patients who receive daily PPI therapy [61]. Maintenance therapy for GERD is recommended at the lowest effective dose. Evidence from randomized controlled trials demonstrate that subjects treated with an H2RA as maintenance are twice as likely to have recurrent esophagitis as those treated with a PPI. However, among patients with NERD, on-demand regimens may be effective [61].

### 8.5. Issues with Chronic PPI Therapy

Proton pump inhibitors are generally well tolerated but there are reports of minor side effects such as headache, diarrhea, and abdominal pain [75, 76]. In general, these occur in about 1–4% of patients and resolve when the treatment is discontinued. Over the short term, PPIs are safe.

The long-term safety of PPIs is not completely understood. Some safety issues have been raised, although most of these have been in epidemiologic, case-control studies. Epidemiologic data are useful in looking for associations, which of course, should not be confused with causality.

Proton pump inhibitors cause hypergastrinemia in response to acid suppression. Enterochromaffin-like cell (ECL), hyperplasia, and carcinoid tumors have been described in rats [77], raising a safety concern in humans. However, several studies in humans did not show similar lesions [78–81]. The associations of fractures of hip, wrist, forearm, and other sites appear weak and only slightly higher than the risks in control populations matched for age [82–85]. However, there is an urgent need for careful prospective studies of the effects of PPIs on bone metabolism and for epidemiological studies carefully designed to minimize confounding by various clinical variables. The risks of Clostridium difficile colitis, other enteric infections, small bowel bacterial overgrowth, and possibly spontaneous bacterial peritonitis also appear increased [86–88]. Impaired gastric secretion may adversely affect the absorption of various nutrients but their clinical impact is still ill-defined [89]. Interaction of PPI with other drugs has assumed tremendous importance recently. Co-therapy with clopidogrel and low-dose PPI therapy is widely used to minimize the risk of serious gastrointestinal bleeding, particularly in high-risk patients, so a balancing of risks in the individual patient is appropriate. Although the FDA has recently promulgated some cautionary statements, these remain controversial [90].

The true importance of these concerns regarding the safety of long-term PPI use can only be estimated from prospective and where possible randomized studies designed solely to measure safety, with minimal confounding.

## 9. Newer Treatments

Acid-suppressive therapy currently forms the mainstay of treatment for GERD, and PPI is the drug of choice in this regard [51]. However, a substantial proportion of patients diagnosed with GERD continue to experience symptoms despite PPI treatment [70, 91], and 22% of PPI users report taking additional over-the-counter (OTC) medicines to control their symptoms [71].

Transient lower esophageal sphincter relaxation (TLESR) is an important factor behind the occurrence of reflux, and preclinical studies have identified gamma aminobutyric acid (GABA) type B receptor (GABA_B_) agonists and metabotropic glutamate receptor 5 (mG1uR5) modulators as candidate drugs for modifying TLESR. Baclofen is an example of the former, while ADX10059 is an example of the latter. Both drugs reduce the incidence of TLESR but poor tolerability is the key issue with these drugs [92].

Potassium-competitive acid blockers (P-CAB) are a group of acid-suppressive drugs that inhibit gastric H^+^K^+^-ATPase (proton pump) reversibly rather than irreversibly. Whereas the PPIs covalently and irreversibly block the proton pump of the gastric parietal cell [93, 94], P-CABs exert their effect by reversible, potassium-competitive binding at, or near, the potassium-binding site on the proton pump [95]. Unfortunately randomized, double-blind trials have not demonstrated any superiority of P-CABs over PPIs [95, 96]. However, there are two other molecules in the same group that are showing some promise [97, 98].

5-hydroxytryptamine type 4 (5-HT_4_) receptor agonists increase gastric smooth muscle contractility. This receptor is a potential new target in GERD [99]. Drugs in this class include cisapride, monsapride, and togaserod (which is also used in the treatment of constipation and irritable bowel syndrome). However, safety issues have limited their usefulness in contemporary clinical practice [100, 101]. ATI-7505 is a cisapride analogue that is currently undergoing trial [102].

Known modulators of visceral pain such as tricyclic antidepressants and selective serotonin reuptake inhibitors (SSRIs) may present an attractive option for GERD patients. A randomized double-blind trial assessing the efficacy of pain modulation with nortriptyline, a tricyclic antidepressant in patients with GERD who have failed to respond to standard-dose PPI therapy is currently on-going [103].

## 10. Surgery

To address the chronic and relapsing nature of GERD, two treatment options are available and these are long-term medication and surgery. The advantages and disadvantages of long-term medical treatment and surgery are shown in Table 2 [104]. A multicentre study which compared optimized esomeprazole therapy and standard laparoscopic antireflux surgery (LARS) in patients with GERD demonstrated that both approaches are equally effective as most patients achieve and remain in remission at 5 years [105].

### 10.1. Indications for Surgery

1. Failed medical management (inadequate symptom control, severe regurgitation not controlled with acid suppression, or medication side effects).
2. Patients who opt for surgery despite successful medical management (due to quality of life considerations, life-long need for medication intake, expense of medication etc.)
3. Complications of GERD (Barrett's esophagus, peptic stricture) [106, 107].
4. Extraesophageal manifestations (asthma, hoarseness, cough, chest pain, and aspiration) [108–111]. The coexistence of Barrett's esophagus with reflux symptoms is considered by many as clear indication for antireflux surgery [112].

Over the past 50 years, surgery for GERD has evolved from an open to a laparoscopic procedure and recently to a new incisionless procedure called transoral incisionless fundoplication. The most common procedure is Nissen fundoplication, which can be open or laparoscopic. Fundoplication can involve a complete (360 degrees) or partial (varying degrees) wrap of the LES with a portion of the stomach, thereby increasing the LES pressure. In the era of open antireflux surgery, symptom response rates of 80–90% were reported [113, 114]. Even at that, many patients avoided it because of high morbidity. With the introduction of laparoscopic techniques, there has been an exponential growth in the number of antireflux operations. The advantages include fewer incisional hernias, shorter hospital stay, less pain, quicker return to work, and fewer defective wraps at follow-up endoscopy [115].

Complications of fundoplication include persistent dysphagia, inability to belch and vomit, epigastric fullness, bloating and postprandial pain, temporary swallowing discomfort, and sometimes intense flatus [116]. Inability to belch, epigastric fullness, bloating, and flatus constitute the syndrome of “gas bloat”.

Endoluminal fundoplication is a new, modified version of open or laparoscopic fundoplication which accesses the stomach through the mouth, thereby eliminating the need for incisions.

## 11. Overlap of GERD with Other Gastrointestinal Disorders

Patients with NERD have other functional gastrointestinal symptoms, such as functional dyspepsia and irritable bowel syndrome (IBS), with a frequency higher than that observed in most studies of erosive reflux disease [117–119]. A common denominator may well be visceral hypersensitivity [120]. The NERD patient group incorporates subgroups which differ significantly in terms of presentation, pathophysiology, and management. Patients with functional heartburn are more likely to have psychopathology, similar to functional dyspepsia patients [121]. Abdominal symptoms appear to be independent predictors of severity of reflux symptoms in NERD patients when compared to control subjects who do not have such symptoms [122]. The significance of this overlap is still a subject of serious research, and it does appear that more revisions await the classification of functional gastrointestinal disorders.

## 12. Conclusion

In conclusion, GERD is one aspect of gastroenterology that has undergone tremendous innovations in the last 30–40 years and is still an area of intensive research. There have been innovations in the definition, classification, diagnosis, clinical course, and management of GERD. Nonerosive reflux disease (NERD) is the variant of GERD that affects over 60% of patients with GERD and it is not only more heterogeneous than erosive esophagitis but has a different pathophysiology and response to standard medical therapy. Because GERD is a chronic, relapsing disease, patients have to be managed with either long-term medical treatment or surgery after a thorough analysis of the pros and cons of each modality. A number of issues remain unresolved about GERD and it is hoped that the next couple of years would come with more discoveries in this important disease.

## Figures and Tables

**Table 1:** The Montreal definition of GERD and its constituent syndromes [1].

Esophageal syndromes
Syndromes with symptoms
(i) Typical reflux syndrome
(ii) Reflux chest pain
Syndromes with esophageal injury
(i) Reflux esophagitis
(ii) Reflux stricture
(iii) Barrett's esophagus
(iv) Esophageal adenocarcinoma

Extraesophageal syndromes

Established associations
(i) Reflux cough syndrome
(ii) Reflux laryngitis syndrome
(iii) Reflux asthma syndrome
(iv) Reflux dental erosion syndrome
Proposed associations
(i) Pharyngitis
(ii) Sinusitis
(iii) Idiopathic pulmonary fibrosis
(iv) Recurrent otitis media

**Table 2:** Potential advantages and disadvantages of medical therapy and antireflux surgery in the management of chronic gastroesophageal reflux disease [104].

Medical	
Advantages	
(i) Noninvasive	
(ii) Simple and easy to use	
(iii) Reproducible effect	
(iv) Very effective on symptoms and lesions of GERD	
(v) Excellent tolerance and safety profile of PPI	
(vi) Relatively cheap especially since the development of PPI generics	
Disadvantages	
(i) Does not correct underlying pathophysiological mechanisms	
(ii) Continuous maintenance therapy frequently required to control the disease	
(iii) Persistence of symptoms in at least 10% of patients	
(iv) Rare side effects and potential drug-drug interactions	

Surgery	

Advantages	
(i) The only treatment capable of physically controlling reflux	
(ii) Very effective (improved quality of heartburn control, reduction of regurgitation, better sleep pattern, increased activities and exercise, etc.)	
(iii) Avoids the need to take medication	
(iv) Psychological effects of not having chronic disease	
(v) Particular clinical groups of cystic fibrosis, lung transplant, and congenital hernia	
Disadvantages	
(i) Invasive	
(ii) Small risk of mortality	
(iii) Measurable postoperative mortality	
(iv) Recurrence is possible	
